# Reversal of Lipid Metabolism Dysregulation by Selenium and Folic Acid Co-Supplementation to Mitigate Pathology in Alzheimer’s Disease

**DOI:** 10.3390/antiox11050829

**Published:** 2022-04-24

**Authors:** Zhong-Hao Zhang, Xian-Chun Cao, Jia-Ying Peng, Shao-Ling Huang, Chen Chen, Shi-Zheng Jia, Jia-Zuan Ni, Guo-Li Song

**Affiliations:** 1 Shenzhen Key Laboratory of Marine Bioresources and Ecology, College of Life Sciences and Oceanography, Shenzhen University, Shenzhen 518000, China; zzh@szu.edu.cn (Z.-H.Z.); 2170257221@email.szu.edu.cn (X.-C.C.); 2100251030@email.szu.edu.cn (J.-Y.P.); 1900251027@email.szu.edu.cn (S.-L.H.); 2120180422@email.szu.edu.cn (C.C.); 1950253001@email.szu.edu.cn (S.-Z.J.); jzni@szu.edu.cn (J.-Z.N.); 2 Shenzhen Bay Laboratory, Shenzhen 518000, China; 3 Shenzhen-Hong Kong Institute of Brain Science-Shenzhen Fundamental Research Institutions, Shenzhen 518000, China

**Keywords:** selenium, folate, selenoprotein, Alzheimer’s disease, lipid metabolism, homocysteine

## Abstract

Aberrant lipid metabolism is reported to be closely related to the pathogenesis of neurodegenerative diseases, such as Alzheimer’s disease (AD). Selenium (Se) and folate are two ideal and safe nutritional supplements, whose biological effects include regulating redox and homocysteine (Hcy) homeostasis in vivo. Here, to achieve effective multitarget therapy for AD, we combined Se and folic acid in a co-supplementation regimen (Se-FA) to study the therapeutic potential and exact mechanism in two transgenic mouse models of AD (APP/Tau/PSEN and APP/PS1). In addition to a reduction in Aβ generation and tau hyperphosphorylation, a restoration of synaptic plasticity and cognitive ability was observed in AD mice upon Se-FA administration. Importantly, by using untargeted metabolomics, we found that these improvements were dependent on the modulation of brain lipid metabolism, which may be associated with an antioxidant effect and the promotion of Hcy metabolism. Thus, from mechanism to effects, this study systematically investigated Se-FA as an intervention for AD, providing important mechanistic insights to inform its potential use in clinical trials.

## 1. Introduction

Alzheimer’s disease (AD) is the fifth leading cause of death for people over 65 years old [1]. Starting with memantine (an N-methyl-D-aspartate (NMDA) receptor antagonist), the first noncholinergic agent used for the treatment of moderate to severe AD patients, more than 100 disease-modifying therapies (DMTs) for AD are currently under investigation in clinical trials [2]. However, no approved treatment can reverse or arrest the progression of AD. Even the positive findings on aducanumab (a monoclonal antibody targeting Aβ oligomers) still need further support to confirm the effectiveness of this treatment for future clinical applications [3]. Encouragingly, great progress has also been made in understanding the pathology of AD, as a plethora of complex, progressive, interactive processes that drive degradation [4], suggesting that the existing single-target strategy is not ideal for AD therapy.

Given the complex intercorrelation among Aβ, tau and other AD-related pathologies [5,6], there is an urgent need to explore multitarget therapies to address cognitive impairment and a variety of AD pathologies. In recent years, it has become popular to repurpose natural nutrients or organic substances, which have been demonstrated to be antioxidants or involved in epigenetic mechanisms [7]. Selenium (Se) is an essential trace element with strong antioxidant capacity in vivo, and various Se compounds have shown positive effects in AD mice, manifested mainly by mitigation of tau pathology, restoration of synaptic deficits and amelioration of cognitive decline through the regulation of autophagic pathway and synaptic receptors [8,9,10]. Folate is integral to one-carbon metabolism, facilitating the conversion of homocysteine (Hcy) to methionine (Met), and the correlation of high blood Hcy levels with AD suggests that folate has potential in AD therapy [11]. Folic acid supplementation has been shown to inhibit Aβ accumulation and tau phosphorylation in an AD mouse model [12,13].

The strategy of medical dietary supplements has always occupied a place in the field of drug development because it is readily able to achieve synergistic effects and lower the effective threshold for administration, thus, reducing the potential side effects. A dietary supplement called Souvenaid, composed of omega-3 fatty acids, minerals and vitamins, has been shown to cause improvements in patients at early stages of AD, in multiple randomized controlled trials (RCTs) [14,15]. Additionally, several combinations containing folic acid or selenium were used to alleviate the clinical symptoms of AD patients in related RCTs, such as probiotic and selenium co-supplementation [16] and the combination of oral folic acid and vitamin B12 [17]. However, due to the complexity of the composition of medical dietary supplements, their definite targets and exact mechanism in AD are unclear, which may limit their widespread recognition in the treatment of AD.

In this study, we administered a combination of Se and folic acid (Se-FA) to two classic AD mouse models (APP/Tau/PSEN and APP/PS1) as a dietary supplement. Se-related selenoproteins and selenoenzymes and folic-acid-regulated Hcy metabolism were detected. In addition, we comprehensively investigated AD-related pathologies to observe the multitarget effect of Se-FA. More importantly, we demonstrated that the modulatory effect of Se-FA on lipid metabolism (which plays a crucial role in its mechanism of action) can almost completely reverse pathological changes in metabolite levels in the brains of AD mice.

## 2. Materials and Methods

### 2.1. Mice and Diet

First, 3×Tg-AD (B6; 129-Psen1tm1MpmTg (APPSwe, tauP301L) 1Lfa/J) and its control (NTg) mice (B6;129SF2/J) as described previously [18] were purchased from the Jackson Laboratory. The uniformity of their reproduction generation and parental mating time was strictly controlled in this study. Further, 2×Tg-AD (B6; C3-Tg (APPswe, PSEN1dE9)) and its control (NTg) mice (B6; C3F1/J) as described previously [19] were purchased from the Model Animal Research Center of Nanjing University. The mice used in this study were littermates. The Se-FA co-supplements consist of selenium yeast (Hua Xin Pharmaceutical) and folate. AD mice were fed diets with normal-dose Se-FA (Se 3 μg/g and folate 36 μg/g), low-dose Se-FA (Se 1.5 μg/g and folate 18 μg/g) or a control diet (common diet) in fodder for 12 weeks, starting at 4 months of age. All the mice were housed 4–7 per cage in a pathogen-free mouse facility with ad libitum access to food and water on a 12 h light/dark cycle. The levels of VB6, VB12 and other vitamins in the co-supplements were below the lower limit of detection (Table 1) and far less than the content in the normal feed (Table 2). All experiments were performed in compliance with guidelines approved by the Animal Ethical and Welfare Committee of Shenzhen University (Permit Number: AEWC-20140615-002).

### 2.2. Se Levels

As previously described [20], the levels of Se in Se-yeast, drinking water, food, hippocampi, cortices, and other tissues were measured by atomic fluorescence spectrometry (AFS-920; Beijing Gitian Instruments, Beijing, China). A standard Se solution (GBW(E) 080215, 100 pg/mL) was obtained from the National Standard Material Research Center (Beijing, China). Five samples were randomly collected from each group.

### 2.3. ELISA and Automated Chemistry Analyzer

The supernatant of brain homogenates was prepared as previously described [10]. The levels of Hcy, VB6, VB12, folate, Met, MS, MDA, ApoE, ApoJ and Aβ1-42 in serum or brain homogenates were estimated using specific ELISA kits, as shown in Table 3, according to the manufacturer’s instructions. The activity levels of GPx and TrxR in the brain homogenates were measured using a GPx assay kit [21] and TrxR assay kit [20], respectively. Levels of total TC, TG, LDL-C and HDL-C in serum were measured by using an automated chemistry analyzer (Icubio, Shenzhen, China) and corresponding kit (Table 3).

### 2.4. Immunoblotting and Immunostaining

Total proteins were extracted in RIPA buffer from tissues or cells, and their concentrations were determined using a BCA Protein Assay (Thermo, Waltham, MA, USA). After boiling, 10–15 μg of protein samples was separated on 10–15% sodium dodecyl sulfate–polyacrylamide gels and transferred to PVDF membranes (Millipore, Burlington, MA, USA), followed by incubation with the primary antibodies at 4 °C overnight and HRP-conjugated secondary antibodies (Table 3) for 1 h at 37 °C. Membranes were visualized using an enhanced chemiluminescence (ECL) kit (FD Neurotechnologies, Irvine, CA, USA), and band intensities of immunoreactive bands were quantified by Fiji (National Institutes of Health, USA).

Fresh mouse brains were fixed with 4% PFA overnight at 4 °C and then transferred to 30% sucrose solution. Brain sections (20 μm) were cut on glass slides and then permeabilized with PBS containing 0.2% Triton X-100 for 10 min at room temperature. For staining and imaging, sections or cells were blocked with 5% goat serum and were then incubated with primary antibodies, followed by incubation with Alexa Fluor fluorescent dye-conjugated secondary antibodies (Table 3). Finally, sections were imaged using fluorescence microscopy (Zeiss, Jena, Germany).

### 2.5. Untargeted Metabolomics by LC–MS

The LC–MS experiment and analyses of untargeted metabolomics were performed as described previously [22]. Briefly, the cortex sample underwent a series of complex processing steps, and then a Dionex Ultimate 3000 RS UHPLC system (Thermo Fisher Scientific, Waltham, MA, USA) was used to analyze the metabolic profile. The acquired LC–MS raw data were analyzed by progenesis QI software (Waters Corporation Milford, Milford, MA, USA). PCA and (O) PLS DA were carried out to visualize the metabolic alterations among experimental groups. The differential metabolites were selected on the basis of the combination of a statistically significant threshold of variable influence on projection (values obtained from the OPLS-DA model and *p* values from a two-tailed Student’s t test on the normalized peak areas, where metabolites with VIP values larger than 1.0 and *p* values less than 0.05 were considered differential metabolites). Heatmaps and hierarchical cluster analyses were conducted using MeV version 4.6.0. Cytoscape software package version 3.2.0 (National Institute of General Medical Sciences, Bethesda, MD, USA) and the KEGG database was used to plot the correlation networks and identify metabolic pathways.

### 2.6. Electrophysiological Analysis

For LTP recording, experiments were performed as previously reported [9]. In brief, 7-month-old mice were anesthetized with isoflurane and decapitated, and brains were quickly isolated and placed into ice-cold artificial cerebrospinal fluid (aCSF) continuously bubbled with carbogen (95% O_2_/5% CO_2_) and cut into 300 mM slices on a vibrating blade microtome (Leica VT1200). Slices were transferred to a holding chamber at 32 °C for 2 h in aCSF with carbogen. fEPSP values were recorded using an MED64 multichannel recording system (Alpha Med Science, Osaka, Japan). For each slice, the baseline stimulus intensity was set at −10 μA every 20 s for 30 min. LTP was induced with a conditioning stimulus consisting of three theta burst trains (ten 5-Hz series of four 100-Hz pulses each, 40 s apart).

### 2.7. Behavioral Tests

The Morris water maze was conducted as previously described with modifications [10]. Each mouse was given three training trials per day for 4 consecutive days, during which the platform was left in the same position. The time taken to reach the platform (escape latency) was measured. A probe trial without a platform was performed 24 and 72 h after the last trial of the hidden platform test. In a 60 s search time, the time spent in each quadrant and the number of times the original platform location was crossed were recorded. Mice were tracked by a camera connected to a video recorder and a computerized tracking system (Smart V3.0, RWD, Shenzhen, China). The Y-maze is classically used to assess short-term memory by allowing mice to explore all three arms of the maze [23]. In the test, each mouse was allowed to explore the three arms for 3 min, and the number and order of entries were recorded.

### 2.8. Statistical Analysis

The data were analyzed using GraphPad Prism software and are expressed as the mean ± SEM (* *p* < 0.05, ** *p* < 0.01, *** *p* < 0.001 and **** *p* < 0.0001). Student’s *t* test was used for simple pairwise comparisons. For multiple comparisons, one-way or two-way ANOVA followed by Dunnett’s multiple comparison test were used.

This section may be divided by subheadings. It should provide a concise and precise description of the experimental results, their interpretation, as well as the experimental conclusions that can be drawn.

## 3. Results

### 3.1. Se-FA Reduces Hcy Levels by Regulating the Hcy-Met Cycle

**Figure 1:**
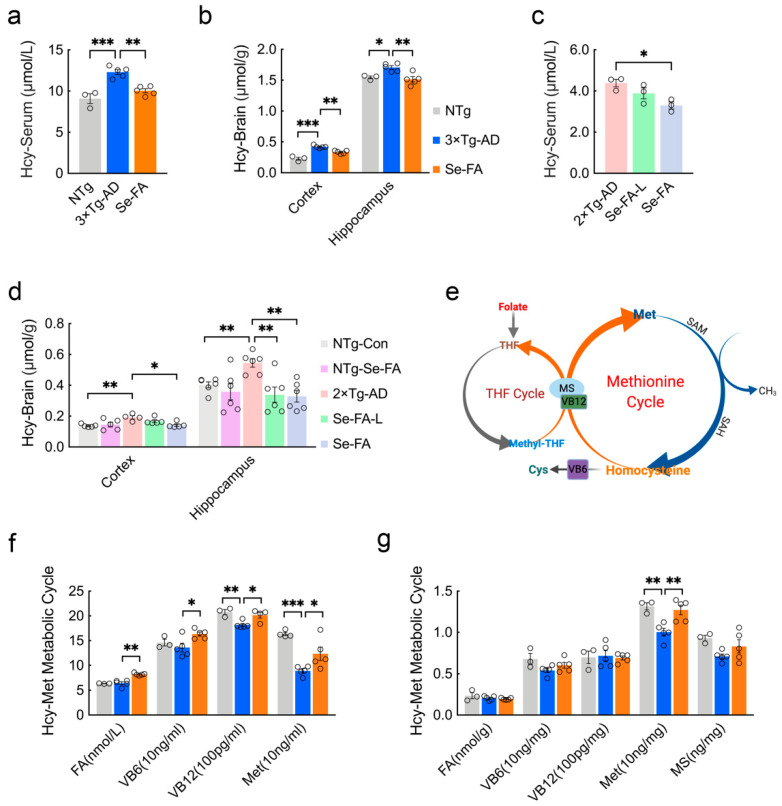
Se-FA reduces Hcy levels and increases Met levels in 3×Tg- and 2×Tg-AD mice. (**a**–**d**) Hcy levels in the serum (**a**,**c**), cortex and hippocampus (**b**,**d**) of 7-month-old 3×Tg- and 2×Tg-AD mice measured by ELISA (n = 3 or 6 mice). Se-FA: normal-dose Se-FA (Se 3 μg/g and folate 36 μg/g). Se-FA-L: low-dose Se-FA, half of normal-dose Se-FA. (**e**) A pattern of THF and Met cycles in the metabolism of Hcy. (**f**,**g**) Levels of FA, VB6, VB12, Met and MS in the serum (**f**) and cortex (**g**) of 7-month-old 3×Tg-AD mice (n = 3, 4 or 5 mice). All the mice were fed diets with Se-FA or a control diet for 12 weeks. All data are presented as the mean ± SEM. * *p* < 0.05, ** *p* < 0.01, *** *p* < 0.001 as determined by one-way ANOVA followed by Dunnett’s multiple comparison test.

Early studies have found that Hcy elevation is a risk factor for AD, and a high Hcy level is a consequence of folic acid deficiency [24,25]. To assess the effect of Se-FA on Hcy levels in AD, we treated 3×Tg- and 2×Tg-AD mice with Se-FA for 3 months. It is well known that the cortex and hippocampus are the major regions affected by AD-related pathology. Consistent with the results of others [26], Hcy levels in the serum, cortex and hippocampus of AD mice were significantly increased compared with NTg mice. Se-FA administration significantly lowered the levels of Hcy, and a similar effect was shown in the hippocampus of 2×Tg-AD mice treated with low-dose Se-FA (Figure 1a–d). Intriguingly, no significant changes were observed in the brains of Se-FA-treated NTg mice (Figure 1d).

There are two cycles in the metabolism of Hcy, and folate participates in the circulation in the form of tetrahydrofolate (THF). As Figure 1e shows, methyl transfer from methyl-THF to homocysteine is catalyzed by vitamin B12 (VB12)-dependent Met synthase (MS), producing Met and THF. Then, Met is catalyzed into S-adenosyl-methionine (SAM) and S-adenosylhomocysteine (SAH), in turn, and finally, Hcy. In addition, vitamin B6 (VB6)-dependent cystathionine synthase catalyzes the conversion of homocysteine to cysteine, which is a precursor of glutathione (Figure 1e). To assess the regulation of Hcy metabolism by Se-FA, we further quantified the levels of FA, VB6, VB12 and Met in the serum of 3×Tg-AD mice and found significant increases in all of them, upon treatment with Se-FA (Figure 1f). In the brain, Se-FA significantly reversed the lower level of Met in AD mice but was limited to the cortical area (Figure 1g). In 2×Tg-AD mice, the level of Met was also markedly elevated in the serum of Se-FA-treated AD mice (Figure S1a). There were no significant differences in the levels of FA, VB6, VB12 and MS in the cortex and hippocampus among all groups of 3×Tg- and 2×Tg-AD mice (Figure 1g and Figure S1a–e). Collectively, these data suggest that Se-FA enhances the metabolic cycle from Hcy to Met, thereby reducing the elevated levels of Hcy in AD mice.

### 3.2. Se-FA Increases Se Levels, Selenoenzyme Activity and Selenoprotein Expression

**Figure 2:**
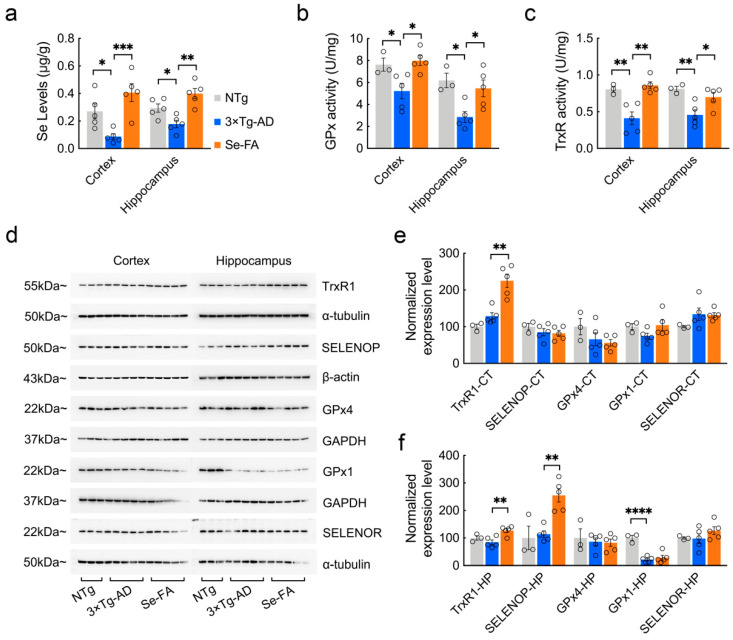
Detection of Se levels, selenoenzyme activity and selenoprotein expression in the brains of 7-month-old AD mice upon Se-FA administration. (**a**) Se levels in the cortex and hippocampus of NTg, 3×Tg-AD and Se-FA-treated mice were measured by atomic fluorescence spectrometry (n = 5 mice). (**b**,**c**) The activity of GPx (**b**) and TrxR (**c**) in the brains of 3×Tg-AD mice was detected using specific assay kits (n = 3 or 5 mice). (**d**) Levels of trxR1, SELENOP, GPx4, GPx1 and SELENOR proteins in the cortex and hippocampus of 3×Tg-AD mice were analyzed by immunoblotting. (**e**,**f**) Quantitation of the protein levels in d ((**e**) cortex; (**f**) hippocampus; n = 3 or 5). α-Tubulin, β-actin or GAPDH was used as a loading control. All the mice were fed diets with Se-FA or a control diet for 12 weeks. All data are presented as the mean ± SEM. * *p* < 0.05, ** *p* < 0.01, *** *p* < 0.001, **** *p* < 0.0001 as determined by one-way ANOVA followed by Dunnett’s multiple comparison test.

We previously demonstrated that Se levels in the cortex and hippocampus of AD mice were significantly lower than those of NTg mice [27]. Here, compared with NTg mice, 7-month-old 3×Tg-AD mice also showed a significant decrease in Se levels in the brain, and Se-FA administration significantly increased Se levels, which were even higher than those in NTg controls (Figure 2a). Se exerts its biological functions mainly through selenoproteins and selenoenzymes. Consistent with the Se levels, we observed reductions in the activity of GPx and TrxR (the two major selenoenzymes in the brain) in AD mice, and Se-FA significantly enhanced their activity in the cortex and hippocampus (Figure 2b,c).

According to existing evidence on the function and distribution of selenoproteins in cells [28], several antioxidative and endoplasmic reticulum (ER)-resident selenoproteins (TrxR1, SELENOP, GPx4, GPx1, SELENOR, SELENOK, SELENOM, SELENOS, SELENON, SELENOT) were detected upon Se-FA administration. Immunoblot analysis of these selenoproteins revealed a significant improvement in TrxR1 expression levels in the brains of Se-FA-treated AD mice (Figure 2d–f). Meanwhile, in the hippocampal area, Se-FA significantly promoted the expression of SELENOP (Figure 2d,f), which is a unique plasma Se transport protein. No significant differences were observed in other selenoprotein levels between the AD controls and Se-FA groups (Figure 2d–f and Figure S1g,f). The above data indicate that Se-FA increases Se levels by promoting the expression of SELENOP and enhances selenoenzyme activity in the brains of AD mice.

### 3.3. Se-FA Reverses Pathological Changes in Metabolism in the Brains of AD Mice

**Figure 3:**
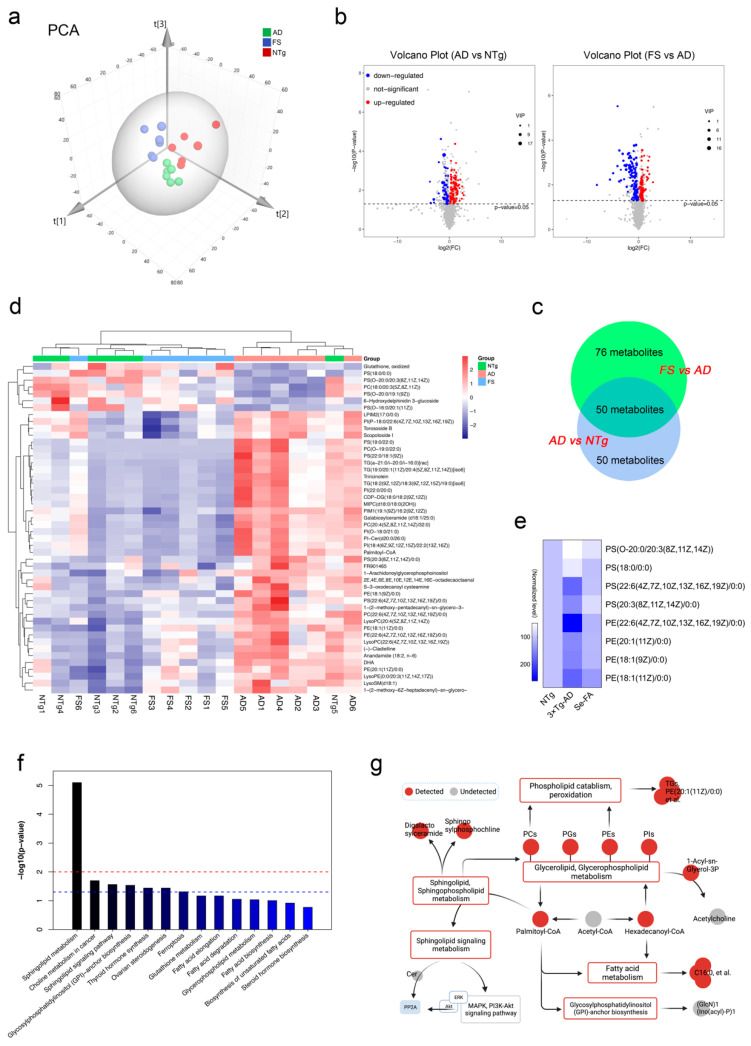
LC–MS untargeted metabolomics analysis reveals altered metabolites and metabolic pathways in the two paired comparisons of AD versus NTg and FS versus AD. (n = 6 mice) (**a**) The PCA score plots obtained in the NTg, AD and FS groups. (**b**) Volcano plot of metabolite alterations in the two paired comparisons: AD versus NTg and FS versus AD. Significantly different proteins (FDR < 0.05 and fold change >2) are colored red (upregulated) and blue (downregulated); others are colored gray. (**c**) Venn diagram with the number of differential metabolites in the two comparisons. (**d**) Clustering heatmap of shared differential metabolites in both comparison groups in AD vs. NTg and FS vs. AD. (**e**) Heatmap of normalized expression levels of metabolites involved in the process of phospholipid peroxidation. (**f**) Kyoto Encyclopedia of Genes and Genomes (KEGG) enrichment analysis of 50 shared differential metabolites. (**g**) The metabolic network of the significantly regulated metabolites and metabolic pathways.

Growing evidence indicates that AD is also a pervasive metabolic disorder [29]. The Hcy-Met cycle, selenoenzymes and selenoproteins are involved in various metabolic processes. To further assess the impact of Se-FA on the brain metabolism of AD, we compared the cortices of NTg-, 3×Tg-AD (AD)- and Se-FA-treated (FS) mice by untargeted metabolomics analysis using LC–MS. Principal component analysis (PCA), shown in Figure 3a, shows the significant differences among these groups, and orthogonal partial least squares discriminant analysis (OPLS-DA) was carried out to visualize the metabolic alterations. In the OPLS-DA model, verified by response permutation testing (RPT), the cumulative parameters of R2Y and Q2 were 0.996 and 0.793 in AD versus NTg (Figure S2b,d), indicating that this model can precisely predict the difference in metabolites in the two comparison groups.

According to the overall contribution of each variable to the OPLS-DA model, we obtained variable importance in the projection (VIP), and the differential metabolites were selected based on VIP values larger than 1 and *p* values less than 0.05. Out of nearly 3068 metabolites identified, 100 metabolites in AD mice were significantly different from those in NTg mice (58 upregulated and 42 downregulated), and there were 126 differential metabolites between the FS and AD groups (46 upregulated and 80 downregulated) (Figure 3b and Figure S2e). As shown in Figure S2f,g of the analysis of hierarchical clustering, the above-selected differential metabolites can clearly distinguish each comparison group and comprehensively exhibit the relations and differences between the groups.

Further analyzing these differential metabolites, we found that 50 shared differential metabolites appeared in both comparison groups in AD vs. NTg and FS vs. AD (Figure 3c). Interestingly, 48 of the 50 differential metabolites in AD mice were reversed by Se-FA treatment, mainly including 38 lipid metabolites classified as glycerophospholipids (GPLs), glycerolipids (GLs), sphingolipids (SLs) and prenol lipids (PLs), in addition to fatty acyls, flavonoids, torososide B and 3 unclassified metabolites (Table S1). The visualized graph of hierarchical clustering analysis of the 48 metabolites revealed that Se-FA significantly reduced approximately 90% of excess lipid metabolites in AD mice and restored the dysregulated lipid metabolites in AD mice to a state close to that of NTg mice (Figure 3d).

To gain insight into the effects of Se-FA on lipid metabolism, the Pearson correlation coefficients between the metabolites were calculated on the basis of the average normalized quantities of metabolites to clarify the latent relations of the top 50 differential metabolites in the comparisons of AD vs. NTg (Figure S3a) and FS vs. AD (Figure S3b). Regarding the functional correlation, highly correlated differential metabolites were connected with lines in networks for a view of the disturbed metabolism of AD and the reversal effect of Se-FA. Most metabolites of phospholipids and SLs had high correlated coefficients, and some of them were prominently related to metabolites of fatty acyls, such as palmitoyl-CoA (Figure S3c,d). In addition, we found that eight GPL metabolites are involved in the process of lipid peroxidation, and Se-FA significantly reversed the alterations of these metabolites in AD, as shown in Figure 3e. Finally, functional annotation and enrichment analysis identified that these differential metabolites were mainly enriched in lipid metabolic pathways involved in SL metabolism, the SL signaling pathway, GPL metabolism, glycosylphosphatidylinositol (GPI) anchor biosynthesis and choline metabolism in the two comparisons (Figure S3e,f), which was consistent with the metabolic pathways enriched by shared differential metabolites (Figure 3f). Notably, disturbance of SL metabolism was the most significant alteration noted in the two paired comparisons.

Combined with the differential metabolite correlations and the enrichment metabolic pathways, a network of metabolic pathways and metabolites was summarized (Figure 3g). Although acetyl-CoA was undetected in metabolites, significant changes in its downstream metabolites palmitoyl-CoA and hexadecanoyl-CoA were observed in AD- and Se-FA-treated mice compared with the control mice. Palmitoyl-CoA, in the center of the network, bridged altered SL and sphingophospholipid (SPL) metabolism, GPL metabolism, GPI-anchor biosynthesis and fatty acid metabolism. The downstream pathway of SL metabolism involved in the regulation of the MAPK and Akt pathways and PP2A was activated by Se-FA treatment. Additionally, hexadecanoyl-CoA participated in modulating GPL metabolism to affect phospholipid (PC, PG, PE and PI) synthesis and metabolism. Due to the regulation of GPL metabolism, the change in the 1-acyl-sn-glycerol-3P metabolite indicated that the acetylcholine synthesis pathway might be regulated. Taken together, the evidence suggests that Se-FA reverses pathological changes in a multitude of lipid metabolites in the brains of AD mice, and SL metabolism, fatty acid metabolism, GPI-anchor biosynthesis and GPL metabolism may be involved in the improving effects of Se-FA on AD pathology.

### 3.4. Se-FA Lowers Blood Lipids and Cerebral Lipid Peroxidation Products

**Figure 4:**
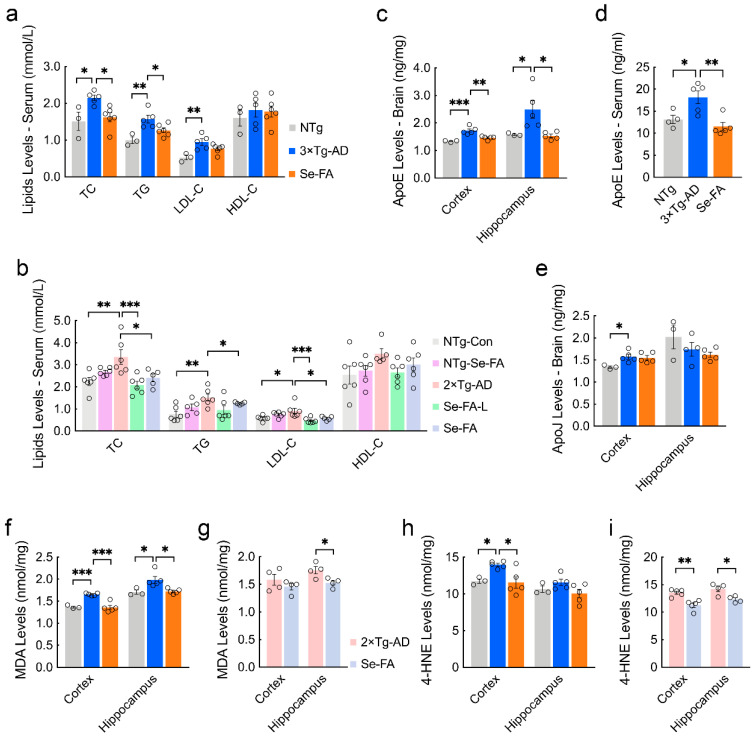
Se-FA reduces the levels of lipids, lipoproteins and MDA in the serum or brain of AD mice. (**a**,**b**) Levels of TC, TG, LDL-C, and HDL-C in the serum of 7-month-old 3×Tg- (**a**) and 2×Tg-AD mice (**b**) measured by an automated chemistry analyzer (n = 3 or 5 mice). (**c**,**d**) ApoE levels in the cortex (**c**), hippocampus (**c**) and serum (**d**) of 7-month-old 3×Tg-AD mice measured by ELISA (n = 3 or 5 mice). (**e**) ApoJ levels in the cortex and hippocampus of 7-month-old 3×Tg-AD mice measured by ELISA (n = 3 or 5 mice). (**f**–**i**) Levels of MDA (**f**,**g**) and 4-HNE (**h**,**i**) in the cortex and hippocampus of 3×Tg- (**f**,**h**) and 2×Tg-AD mice (**g**,**i**) measured by ELISA (n = 3, 4 or 5 mice). All the mice were fed diets with Se-FA or a control diet for 12 weeks. All data are presented as the mean ± SEM. * *p* < 0.05, ** *p* < 0.01, *** *p* < 0.001 as determined by one-way ANOVA followed by Dunnett’s multiple comparison test.

The disturbance of lipid metabolism in the brain reflects the appearance of dyslipidemia in AD mice to a certain extent. In turn, abnormal lipid levels in serum are also an important inducer of disturbed lipid metabolism in the brain. To explore the upstream and downstream mechanisms of lipid metabolism, we employed a chemistry analyzer to detect the levels of blood lipids and found significant increases in the levels of total cholesterol (TC) and triglycerides (TGs) in the serum of 3×Tg- and 2×Tg-AD mice compared with NTg mice (Figure 4a,b). Se-FA treatment significantly reduced the levels of TC and TG, and significant reductions were also shown in low-dose Se-FA-treated mice (Figure 4a,b). The transport of lipids in serum is entrusted to lipoproteins. High-density lipoprotein (HDL) and low-density lipoprotein (LDL) promote cholesterol output and accumulation, respectively. Levels of LDL cholesterol (LDL-C) and HDL cholesterol (HDL-C) (reflecting its corresponding lipoprotein levels) were detected, and AD mice showed a higher level of LDL-C and a decrease upon Se-FA administration. No significant differences were observed in HDL-C levels among NTg-, AD- and Se-FA-treated mice (Figure 4a,b). Similar phenomena were observed in 2×Tg-AD mice treated with both low- and normal-dose Se-FA (Figure 4a,b), suggesting that Se-FA achieves resistance to hyperlipidemia in AD by decreasing the level of LDL.

To determine the relationship between serum lipid levels and brain lipid metabolism, we tested the levels of apolipoprotein E (ApoE) and apolipoprotein J (ApoJ), which are crucial apolipoproteins present in the brain and are involved in binding and transporting blood lipids to the brain for metabolism and utilization [30]. ELISA analysis showed that ApoE levels were significantly elevated in the cortex, hippocampus and serum of AD mice, and significant reductions appeared in Se-FA-treated mice (Figure 4c,d). However, no significant changes in the levels of ApoJ were detected upon Se-FA administration, although its level also increased in the cortex of AD mice (Figure 4e). In addition to the disturbance of lipid metabolism, alterations in the lipid peroxidation process were also observed in the metabolomics data. As the two major metabolic end products of lipid peroxidation in the brain, the levels of malondialdehyde (MDA) and 4-hydroxynonenal (4-HNE) in 3×Tg- and 2×Tg-AD mice were significantly higher than those in NTg mice. Se-FA treatment significantly decreased MDA and 4-HNE levels in the brains of both 3×Tg-AD and 2×Tg-AD mice (Figure 4f–i). Combined, these data demonstrate that Se-FA reduces the levels of blood lipids and ApoE to regulate lipid metabolism and reverse lipid peroxidation in the brain.

### 3.5. Se-FA Attenuates Aβ and Tau Pathologies

**Figure 5:**
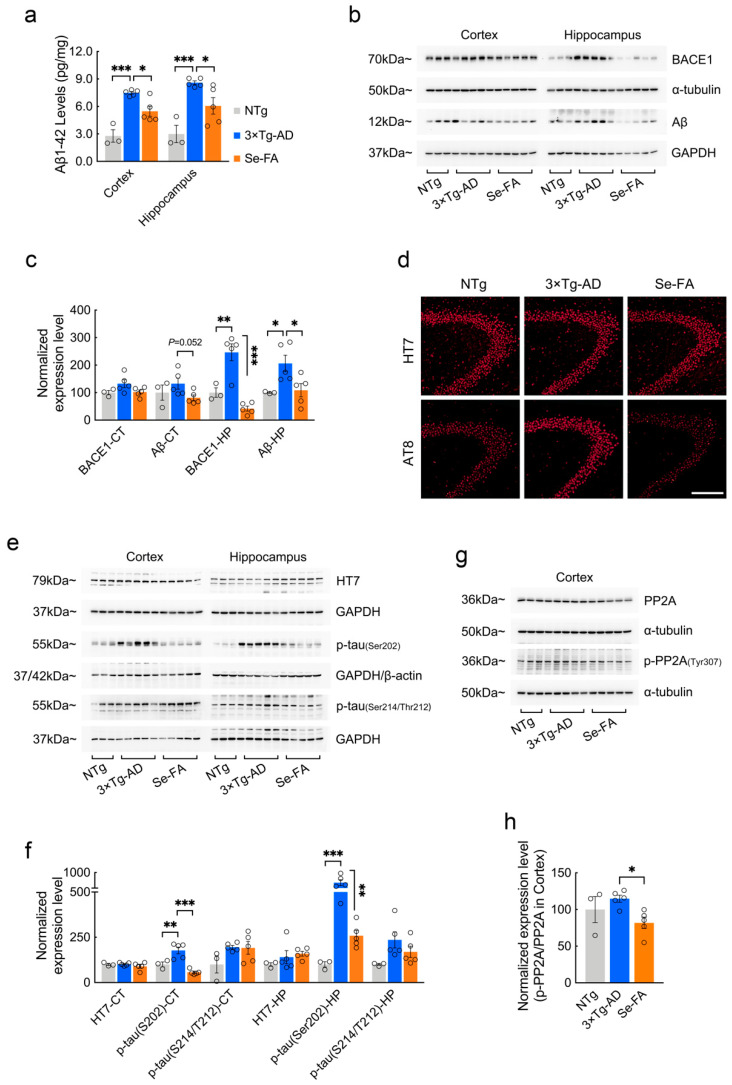
Se-FA inhibits Aβ production and tau hyperphosphorylation in 3×Tg-AD mice. (**a**) Aβ1-42 levels in the cortex and hippocampus of 7-month-old 3×Tg-AD mice measured by ELISA (n = 3 or 5 mice). (**b**) Levels of BACE1 and Aβ oligomers were analyzed by immunoblot in the cortex and hippocampus of 3×Tg-AD mice. (**c**) Quantitation of the protein levels in (**b**) (n = 3 or 5 mice). (**d**) Representative total tau (HT7) and pS202-tau (AT8) immunostaining in the CA3 area of the hippocampus of NTg-, 3×Tg-AD- and Se-FA-treated mice (Scale bar: 50 μm). (**e**) Levels of total tau and p-tau (Ser202 and Ser214/Thr212) in the cortex and hippocampus of experimental mice were analyzed by immunoblotting. (**f**) Quantitation of the protein levels in e (n = 3 or 5 mice). (**g**) Levels of PP2A and p-PP2A (Tyr307) in the cortex of 3×Tg-AD mice were analyzed by immunoblotting. (**h**) Quantitation of the protein levels in g (n = 3 or 5 mice). α-Tubulin, β-actin or GAPDH was used as a loading control. All the mice were fed diets with Se-FA or a control diet for 12 weeks. All data are presented as the mean ± SEM. * *p* < 0.05, ** *p* < 0.01, *** *p* < 0.001 as determined by one-way ANOVA followed by Dunnett’s multiple comparison test.

To evaluate whether the reversal effects of Se-FA administration on lipid metabolism affect AD-related pathology, we further examined Aβ and tau pathology in 3×Tg- and 2×Tg-AD mice upon Se-FA administration. AD mice showed significantly higher levels of Aβ1-42 in the cortex and hippocampus than NTg mice and were significantly decreased in Se-FA-treated AD mice (Figure 5a and Figure S4a). Consistent with the results of the Aβ ELISA, Se-FA also significantly reduced the elevated Aβ oligomer levels in the hippocampus of AD mice (Figure 5b,c). Aβ is produced by the APP cleavage pathway dominated by β-secretase (BACE1). Thus, we measured the level of BACE1 by immunoblot and found that it was significantly upregulated in the hippocampus of 3×Tg-AD mice. A significant decrease in BACE1 levels was detected in Se-FA-treated mice, indicating that Se-FA inhibits BACE1-mediated APP cleavage to reduce Aβ levels (Figure 5b,c).

Regarding tau pathology, immunofluorescence and immunoblot analysis using the anti-phospho-tau antibody AT8 (pS202-tau) revealed a significant increase in hyperphosphorylated tau in 3×Tg-AD mice and a significant reduction in hyperphosphorylated tau in Se-FA-treated AD mice compared to their corresponding controls (Figure 5d–f). However, no significant differences in the levels of total tau and phosphorylated tau at Ser214/Thr212 were observed among the three groups (Figure 5d–f). According to the findings from metabolomics analysis, PP2A regulates tau dephosphorylation and is one of the crucial kinases involved in SL signaling and metabolism. Here, the ratio of p-PP2A/PP2A that inversely correlated the activity of PP2A in vivo was significantly lower in the cortex of Se-FA-treated mice than in the cortex of AD mice (Figure 5g,h), suggesting that Se-FA reduces tau hyperphosphorylation by enhancing the activity of PP2A. Therefore, Se-FA administration inhibited BACE1-mediated Aβ production and enhanced PP2A-regulated tau dephosphorylation.

### 3.6. Se-FA Rescues Synaptic and Behavioral Deficits in AD Mice

**Figure 6:**
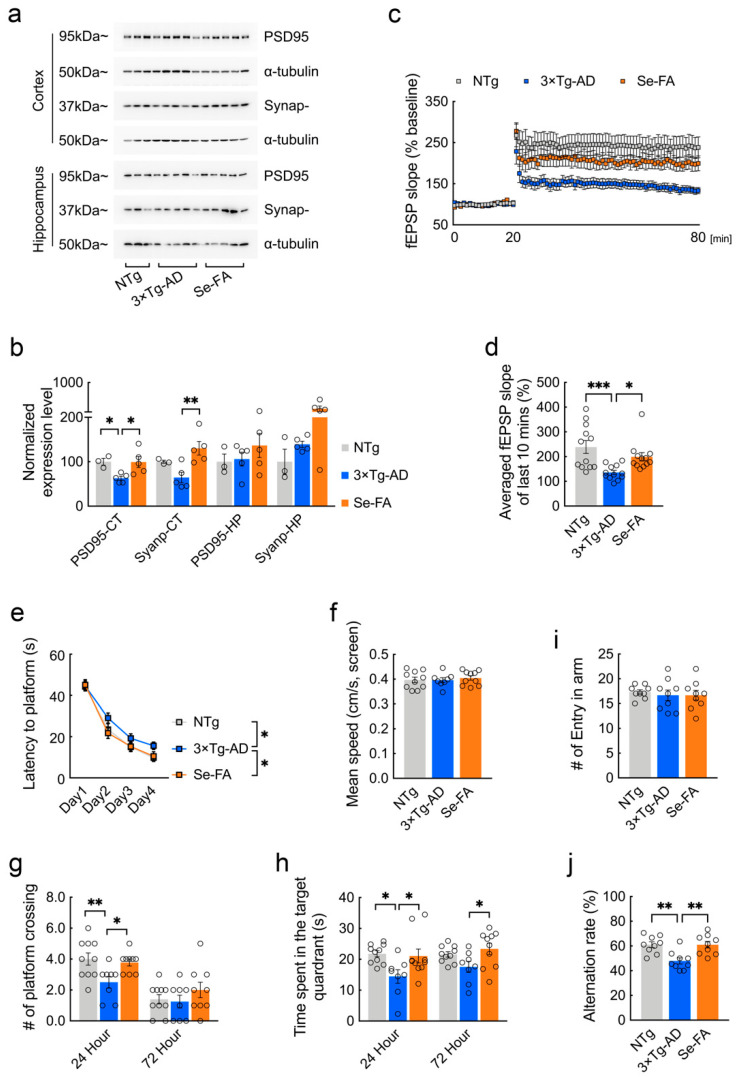
Se-FA restores synaptic plasticity and improves cognitive ability in 3xTg-AD mice. (**a**) Levels of PSD95 and synaptophysin were analyzed by immunoblot in the cortex and hippocampus of mice. α-Tubulin was used as a loading control. (**b**) Quantitation of the protein levels in (**a**) (n = 3 or 5 mice). (**c**) Slope of fEPSPs in response to 100-Hz stimulation in the Schaffer collateral CA1 region of the mice. (**d**) Quantification of the averaged fEPSP slope of the last 10 min in (**c**) (n = 12 sections, 3 sections per mouse). (**e**–**h**) Morris water maze tests were performed in 7-month-old 3×Tg-AD mice (two-way ANOVA followed by Dunnett’s multiple comparison test) (n = 8 or 10 mice). The escape latency time to reach the hidden platform was recorded during the 4-day training (**e**). Swimming speed was recorded during the probe trial (**f**). The probe trial was performed 24 and 72 h after the last trial of a hidden platform task. The number of crossings over the original platform area was recorded (**g**), along with the percentage of search time spent in each quadrant (**h**). (**i**,**j**) The number of arm entries (**i**) and number of correct alternations (**j**) in the Y maze test (n = 9 mice). All the mice were fed diets with Se-FA or a control diet for 12 weeks. All data are presented as the mean ± SEM. * *p* < 0.05, ** *p* < 0.01, *** *p* < 0.001 as determined by one-way or two-way ANOVA followed by Dunnett’s multiple comparison test.

Strong evidence has implicated synaptic failure as a direct contributor to cognitive decline in AD [31,32]. Here, we further analyzed the potential impacts of Se-FA on AD-related synaptic and cognitive deficits. Postsynaptic density protein-95 (PSD95) and synaptophysin are structural proteins located in the postsynaptic and presynaptic membranes, respectively. Immunoblot analysis showed that the levels of the two synaptic proteins were decreased, and that Se-FA significantly reversed the decrease in the cortex of AD mice (Figure 6a,b and Figure S4b,c). To better understand the alteration of synaptic plasticity, we examined long-term potentiation (LTP) in the Schaffer collateral (SC) pathway, with high-frequency stimulation, and found that the LTP impairment of 3×Tg-AD mice was ameliorated upon Se-FA administration (Figure 6c). Additionally, the averaged field excitatory postsynaptic potential (fEPSP) slope of Se-FA-treated mice significantly increased in the last 10 min compared with that of the controls (Figure 6d). Since the change in the last 10 min for LTP is regarded as late-phase LTP accompanied by gene transcription and new protein synthesis [33], these data revealed the restoration effects of Se-FA on synaptic plasticity.

The spatial learning and memory function of experimental mice were evaluated by the Morris water maze and Y maze. In the Morris water maze test, 3×Tg- and 2×Tg-AD mice were less efficient at finding the hidden platform, indicating a deficit in spatial learning ability at 7 months of age. Se-FA rescued this spatial learning deficit, as evidenced by a reduced latency to reach the platform during the 4 days of training (Figure 6e and Figure S4d). In the probe trials conducted 24 and 72 h after the last training, Se-FA-treated AD mice spent significantly more time in the target quadrant and swam more frequently across the original platform area than the controls (Figure 6g,h and Figure S4e,f). A similar effect was observed in the Y maze test, and Se-FA treatment also significantly increased the correct alternation ratio of arm entries, suggesting an improvement in spatial learning ability upon Se-FA administration (Figure 6j). Thus, Se-FA rescues LTP and cognitive deficits in AD mice.

## 4. Discussion

Medical dietary supplementation is a relatively safe and reliable strategy and exerts multitarget effects in therapy. However, its application in clinical practice is limited by its unclear mechanism of action. In this study, selenium and folic acid, which possess strong biological effects [34,35], were used as co-supplementation regimen to evaluate their therapeutic potential in AD. With a combination of biochemical and immunohistological methods, we found that Se-FA significantly alleviated the level of Aβ and tau hyperphosphorylation in the brains of two classic mouse models of AD, suggesting that Se-FA could improve classic AD-related pathology in mice. Moreover, these pathological improvements will directly drive the reversal of synaptic and cognitive deficits. Thus, these data demonstrated the highly effective multitarget effects of Se-FA on AD.

In addition to senile plaques (SPs) and neurofibrillary tangles (NFTs), the AD brain displays a higher accumulation of “adipose inclusions” or “lipoid granules”, suggesting aberrant lipid metabolism [36]. Intriguingly, we found alterations in lipid metabolites in the brains of AD mice, such as GPLs, GLs, SLs, PLs and fatty acyls. Notably, Se-FA reversed the pathological changes in lipids by restoring the shared metabolites to a similar state as NTg mice, which was further suggested by the effects on blood lipids, implying that the regulation of lipid metabolism may be the target of Se-FA in AD treatment.

It has now been determined that abnormal lipid metabolism is implicated in AD pathogenesis [37,38,39]. Lipid rafts are dynamic structures within cell membranes, characterized by combinations of SLs, cholesterol and saturated fatty acids [39]. Many AD-associated proteins are lipid-raft-resident proteins [40]. APP processing within lipid rafts was largely responsible for amyloidogenicity, which is related to lipid composition within lipid rafts [41]. Cholesterol depletion or lowering the levels of SLs decreased the generation of Aβ by disturbances of the association of BACE1 and APP with lipid rafts [42,43,44,45]. Other evidence has shown that the introduction of a GPI anchor, a targeting motif for lipid raft localization, into the BACE1 sequence strongly promotes amyloidogenic processing of APP, further suggesting the key role of lipids in the BACE1-raft association in Aβ generation [41,46]. Here, the decreased levels of major lipid classes (SLs and cholesterol) in Se-FA-treated mice provided a potential mechanism for the inhibition of Aβ production that modulates the biophysical properties of rafts by altering their lipid composition to decrease the association of APP/BACE1 with these lipid microdomains, especially the negative regulation of GPI-anchor biosynthesis by Se-FA, as shown in Figure 3f. Furthermore, the presence of lipid rafts provided a platform in which Aβ conveniently interacts with tau to promote phosphorylated tau [47]. Indeed, Se-FA significantly reduced the levels of tau phosphorylation in the present study. Moreover, PP2A-mediated tau dephosphorylation could be a direct way by which Se-FA improves tau pathology, as the metabolomics analysis data attributed PP2A to the regulation of SLs and its signaling metabolism was affected by Se-FA [48]. Although the intermediate metabolites during this metabolic process were undetected, the enhancement of PP2A activity upon Se-FA administration was determined by subsequent immunoblot analysis.

In addition to lipid composition, the lipid peroxidation process is also an important aspect of lipid metabolism. Our data showed changes in a series of intermediate metabolites of lipid peroxidation and a reduction in MDA and 4-HNE levels in the brains of Se-FA-treated mice, suggesting the effect of Se-FA on lipid peroxidation. Accumulating studies have pointed to a pivotal role of lipid peroxidation in the pathological development of AD. Lipid peroxidation may be upstream of Aβ pathology because it can be detected prior to Aβ deposition in AD mice [49], and lipid peroxidation products increase amyloidogenic APP processing by upregulating BACE1 expression in vivo [50,51]. Similarly, tau phosphorylation, aggregation and filament formation were also found to be affected by lipid peroxidation in neural cells [52,53]. These findings, together with our findings, suggested the role of lipid metabolism regulation in the effects of Se-FA on inhibiting Aβ production and tau hyperphosphorylation. However, the impact of lipid peroxidation on AD is far more extensive. Lipid peroxidation products (4-HNE) can render neurons vulnerable to excitotoxicity by regulating NMDA channel activity [54], indicating a direct effect of lipid peroxidation on synaptic plasticity. In addition, excessive cell death induced by lipid peroxidation may underlie major neurodegeneration [55,56], such as ferroptosis, which also emerged in KEGG enrichment analysis of differential metabolites. Collectively, the reversal alterations of lipid metabolic disturbance play a dominant role in the mechanism of Se-FA to alleviate AD pathology.

As mentioned before, selenium and folic acid play biological roles in vivo in maintaining antioxidant capacity and Hcy levels, respectively. As the core of the one-carbon cycle, Hcy directly affects Met synthesis and metabolism, thereby sustaining normal DNA methylation. Relevant studies have shown that Met deficiency and supplementation can induce molecular abnormalities, including deregulation of lipid metabolism and induction of oxidative and ER stress [57]. In the present study, Se-FA significantly reversed the pathological reduction in Met levels in AD mice, suggesting that the Se-FA-regulated Hcy-Met cycle is an important mechanism to restore lipid metabolism dysregulation. GPx4 can directly reduce phospholipid hydroperoxide, and depletion of GPx4 induces lipid-peroxidation-dependent cell death [58]. Consistent with our results, Se-FA improved antioxidant capacity in the AD brain by enhancing the activity of GPx and TrxR. Meanwhile, the levels of several GPL metabolites involved in the process of lipid peroxidation were restored in the brains of AD mice upon Se-FA administration, suggesting that selenoenzyme is the main target of Se-FA to reduce lipid peroxidation.

Existing studies established that high levels of oxidative stress and Hcy were closely associated with dyslipidemia due to their ability to affect cholesterol and lipoproteins, including HDL, LDL and ApoA1 [59,60,61]. As an upstream phenotype of lipid metabolism disturbance in the brain, the dysregulation of cholesterol and lipoproteins in the blood was also reversed in AD mice upon Se-FA administration. In particular, ApoE, an important lipoprotein, connects lipid metabolism in plasma and the brain, and it has been proven that its genetic polymorphism is a high risk factor for AD. Interestingly, previous studies showed that SELENOP transported Se to the brain and maintained brain Se levels depending on its receptors [62,63], apolipoprotein E receptor 2 (ApoER2), a member of the lipoprotein receptor family. Recently, it has been confirmed that SELENOP interacts with ApoE through the heparin-binding sites of SELENOP [64], prompting a correlation between lipid metabolism and Se transport. Although the underlying mechanism is not yet clear, alterations in the levels of ApoE and SELENOP in Se-FA-treated mice suggested that Se also played a role in lipid metabolism regulation by its incorporation into SELENOP. Thus, our data demonstrated that Se-FA exerted lipid metabolism-regulating effects through a variety of different and interrelated pathways in AD mice.

In conclusion, this study provided strong evidence that the pathology-reversing effects of Se-FA on blood lipid levels and brain lipid metabolism are crucially involved in the reductions in Aβ generation and tau hyperphosphorylation, thereby rescuing synaptic and behavioral deficits. Due to the imperfection of the current metabolomics database, the related intermediate metabolites in lipid metabolism cannot be determined. However, our findings reveal an integral anti-AD mechanism of Se-FA and support further application of Se-FA in AD therapies.

## Figures and Tables

**Table 1:** The concentrations of VB6, VB12 and vitamin E in Se-FA.

Item	Concentration	Unit	Method
Vitamin B12	Not detected, detection limit 1.0	μg/g	GB/T 5009.217-2008
Vitamin B6	Not detected, detection limit 0.02	mg/100g	GB 5009.154-2016
Vitamin E	<0.12	mg/100g	GB 5009.82-2016

**Table 2:** The concentrations of nutrients and trace elements in normal fodder.

Item	Concentration	Unit
Ca	1.19	%
P	0.77	%
Lys	0.93	%
Met + Cys	0.67	%
Arg	1.02	%
His	0.50	%
Trp	0.22	%
Phe + Tyr	1.50	%
Thr	0.78	%
Leu	1.59	%
Ile	0.76	%
Val	0.90	%
VA	10.70	KIU/kg
VD	1.50	KIU/kg
VE	103.05	IU/kg
VK	6.14	mg/kg
VB_1_	16.00	mg/kg
VB_2_	16.03	mg/kg
VB_6_	10.43	mg/kg
VB_3_	89.00	mg/kg
VB_5_	30.09	mg/kg
Folate	7.45	mg/kg
Biotin	0.28	mg/kg
VB_12_	0.03	mg/kg
Choline	1900.00	mg/kg
Mg	0.26	%
K	0.64	%
Na	0.32	%
Fe	180.00	mg/kg
Mn	123.49	mg/kg
Cu	17.80	mg/kg
Zn	56.20	mg/kg
I	0.61	mg/kg
Se	0.16	mg/kg

**Table 3:** Antibodies and kits.

ANTIBODIES			
Reagent	Source	Applications	Identifier
Rabbit anti-BACE1	CST	1:1000	5606
Mouse anti-Aβ	Biolegend	1:2000	SIG-39300
Rabbit anti-APP	Abcam	1:1000	ab32136
Mouse anti-Tau	Invitrogen	1:500	MN1000
Mouse anti-pSTau (Ser202)	Invitrogen	1:500	MN1020
Mouse anti-pSTau (Ser214)	Invitrogen	1:500	MN1060
Rabbit anti-PP2A	CST	1:1000	2038
Rabbit anti-p-PP2A (Tyr307)	ThermoFisher	1:1000	AA1910N
Rabbit anti-PSD95	Abcam	1:5000	ab76115
Rabbit anti-Synaptophysin	CST	1:1000	4329s
Mouse anti-TrxR1	Santa cruz	1:500	sc-28321
Rabbit anti-GPX4	Abcam	1:1000	ab125066
Rabbit anti-GPX1	GeneTex	1:1000	GTX116040
Rabbit anti-SELENOP	Abcam	1:1000	ab109514
Rabbit anti-MSRB1 (SELENOR)	Invitrogen	1:1000	Cat# PA5-77009
Rabbit anti-SELENOK	Sigma	1:1000	HPA-008196
Mouse anti-SELENOM	Santa cruz	1:1000	sc-514952
Mouse anti-SELENON	Santa cruz	1:1000	sc-365824
Rabbit anti-SELENOS	Proteintech	1:1000	15591
Rabbit anti-SELENOT	Acris	1:1000	AP53842PU
Mouse anti-beta-actin	Proteintech	1:10000	66009
Mouse anti-α-tubulin	Proteintech	1:10000	11224
Rabbit anti-GAPDH	Proteintech	1:10000	10494
Goat anti-Rabbit IgG	CST	1:10000	7074
Goat anti-Mouse IgG	CST	1:10000	7076
Anti-mouse Alexa Fluor 594	Abcam	1:1000	ab150116
Anti-rabbit Alexa Fluor 488	Abcam	1:1000	ab150077
**KITS**			
**Reagent**	**Source**	**Identifier**	
HCY ELISA	MLBIO	ml037451	
VB6 ELISA	MLBIO	ml001803	
VB12 ELISA	MLBIO	ml057867	
Folate ELISA	MLBIO	ml002074	
Met ELISA	MLBIO	ml058151	
MS ELISA	MLBIO	Ml672003	
MDA ELISA	MLBIO	ml002001	
Aβ1-42 ELISA	MLBIO	ml002201	
ApoE ELISA	MLBIO	ml002179	
ApoJ ELISA	MLBIO	Ml880235	
GPx activity Kit	Beyotime	S0058	
TrxR activity Kit	COMIN	TRXR-1-W	
TC Assay Kit	iCubio	CHOD-PAP	
TG Assay Kit	iCubio	GPO-PAP	
HDL-C Assay Kit	iCubio	-	
LDL-C Assay Kit	iCubio	-	

## Data Availability

Data is contained within the article.

## Supplementary Materials

The following supporting information can be downloaded at: https://www.mdpi.com/article/10.3390/antiox11050829/s1, Table S1: Reverse metabolite upon Se-FA administration in 3×Tg-AD mice; Figure S1: Levels of FA, VB6, VB12, Met and MS in AD mice; Figure S2–S3: Data of LC–MS untargeted metabolomics analysis; Figure S4: AD pathologies in 2×Tg-AD mice.
