# Supplementary material for: Reversal of Lipid Metabolism Dysregulation by Selenium and Folic Acid Co-Supplementation to Mitigate Pathology in Alzheimer’s Disease
Source: Antioxidants (Basel). 2022 Apr 24;11(5):829. doi: 10.3390/antiox11050829 (PMC9138008; doi:10.3390/antiox11050829)
Supplement: Supplementary file 1 [file antioxidants-11-00829-s001.zip › antioxidants-1665561-supplementary.pdf]

Supporting Information

Table S1. Reverse metabolite upon Se-FA administration in 3×Tg-AD mice

Class	Metabolites	AD vs NTg	FS vs AD
GPL Glycerophosphoserines	PS(O-20:0/20:3(8Z,11Z,14Z))	down	up
	PS(O-20:0/19:1(9Z))	down	up
	PS(18:0/0:0)	down	up
	PS(O-16:0/20:1(11Z))	down	up
	PS(22:6(4Z,7Z,10Z,13Z,16Z,19Z)/0:0)	up	down
	PS(22:0/18:1(9Z))	up	down
	PS(20:3(8Z,11Z,14Z)/0:0)	up	down
	PS(19:0/22:0)	up	down
GPL Glycerophosphoethanolamines	LysoPE(0:0/20:3(11Z,14Z,17Z))	up	down
	PE(22:6(4Z,7Z,10Z,13Z,16Z,19Z)/0:0)	up	down
	PE(20:1(11Z)/0:0)	up	down
	PE(18:1(9Z)/0:0	up	down
	PE(18:1(11Z)/0:0)	up	down
	1-(2-methoxy-pentadecanyl)-sn-glycero-3-phosphoethanolamine	up	down
	1-(2-methoxy-6Z-heptadecenyl)-sn-glycero-3-phosphoethanolamine	up	down
GPL Glycerophosphoinositols	PI(P-18:0/22:6(4Z,7Z,10Z,13Z,16Z,19Z))	up	down
	PI(O-18:0/21:0)	up	down
	PI(22:0/20:0)	up	down
	PI(18:4(6Z,9Z,12Z,15Z)/22:2(13Z,16Z))	up	down
	1-Arachidonoylglycerophosphoinositol	up	down
GPL Glycerophosphocholines	PC(18:0/20:3(5Z,8Z,11Z))	down	up
	PC(O-19:0/22:0)	up	down
	PC(22:6(4Z,7Z,10Z,13Z,16Z,19Z)/0:0)	up	down
	PC(20:4(5Z,8Z,11Z,14Z)/32:0)	up	down
	LysoPC(22:6(4Z,7Z,10Z,13Z,16Z,19Z))	up	down
	LysoPC(20:4(5Z,8Z,11Z,14Z))	up	down

GPL (Others)	PIM1(19:1(9Z)/16:2(9Z,12Z))	up	down
	LPIM2(17:0/0:0)	up	down
	CDP-DG(18:0/18:2(9Z,12Z))	up	down
GL Triradylcglycerols	Triricinolein	up	down
	TG(a-21:0/i-20:0/i-16:0)[rac]	up	down
	TG(19:0/20:1(11Z)/20:4(5Z,8Z,11Z, 14Z))[iso6]	up	down
	TG(18:2(9Z,12Z)/18:3(9Z,12Z,15Z)/ 19:0)[iso6]	up	down
SL Phosphosphingolipids	MIPC(d18:0/18:0(2OH))	up	down
	LysoSM(d18:1)	up	down
	PI-Cer(d20:0/26:0)	up	down
SL Glycosphingolipids	Galabiosylceramide (d18:1/25:0)	up	down
Fatty Acyls	Anandamide (18:2, n-6)	up	down
	FR901465	up	down
	DHA	up	down
	Palmitoyl-CoA	up	down
	S-3-oxodecanoyl cysteamine	up	down
6-Hydroxydelphinidin 3-glucoside (Polyketides)		down	up
(-)-Cladielline (Prenol lipids)		up	down
Torososide B (Organooxygen compounds)		up	down
Glutathione, oxidized (Unclassified)		down	up
Scopoloside I (Unclassified)		up	down
2E,4E,6E,8E,10E,12E,14E,16E-octadecaoctaenal (Unclassified)		up	down

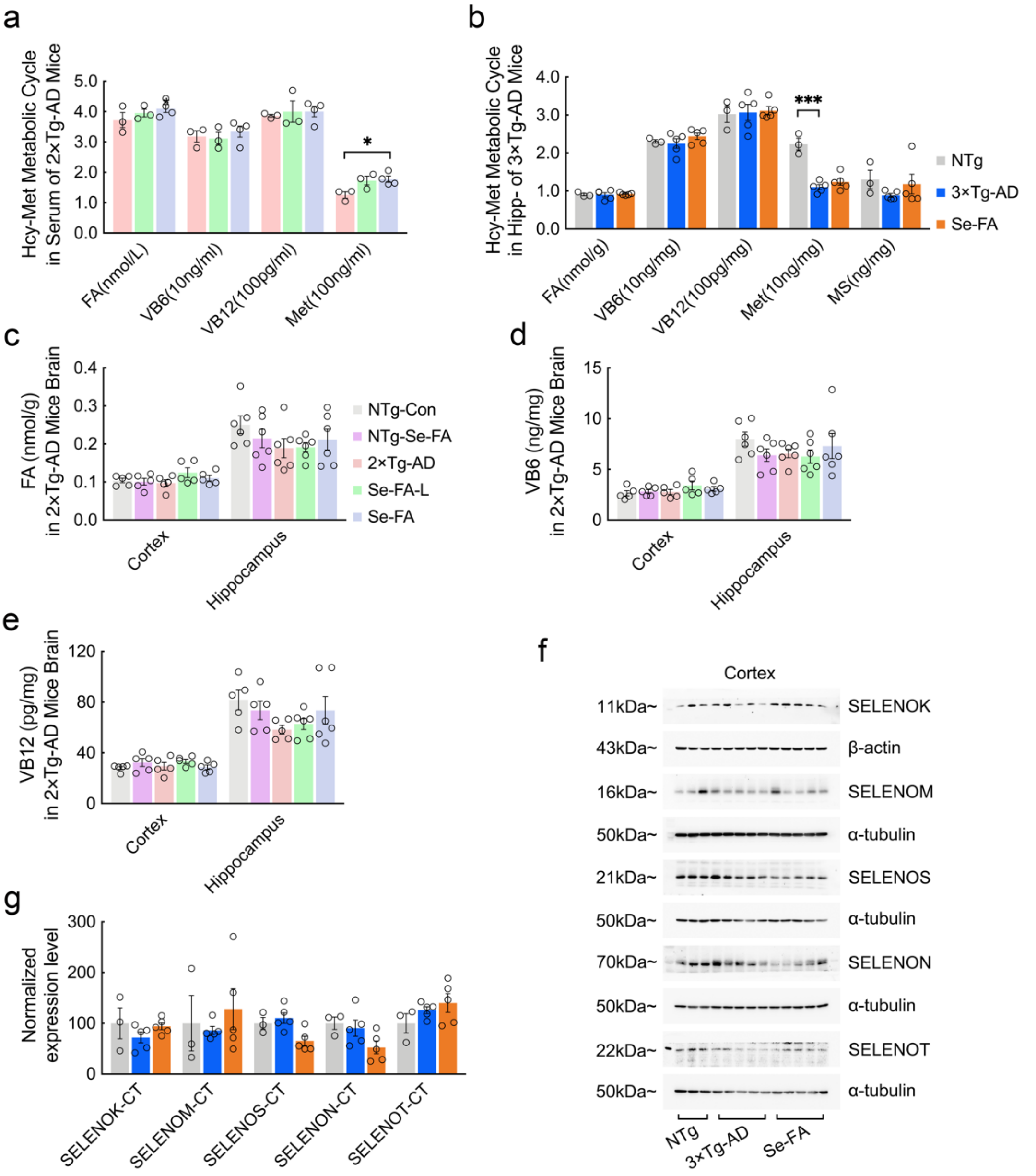

Figure S1 a Levels of FA, VB6, VB12, Met and MS in the hippocampus of 7-month-old 3×Tg-AD mice measured by ELISA (n=3 or 5 mice). **b** Levels of FA, VB6, VB12, and Met in the serum of 7-month-old 2×Tg-AD mice (n=3 or 4 mice). **c-e** Levels of FA, VB6 and VB12 in the cortex and hippocampus of 2×Tg-AD mice (n=3 or 5 mice). **f** SELENOK, SELENOM, SELENOS, SELENON and SELENOT proteins in the cortex of 3×Tg-AD mice were analyzed by immunoblotting. **g** Quantitation of the protein levels in **f**. α-Tubulin or β-actin was used as a loading control. All data are presented as the mean ± SEM. **p*<0.05, ***p*<0.01, ****p*<0.001 as determined by one-way ANOVA followed by Dunnett's multiple comparison test.

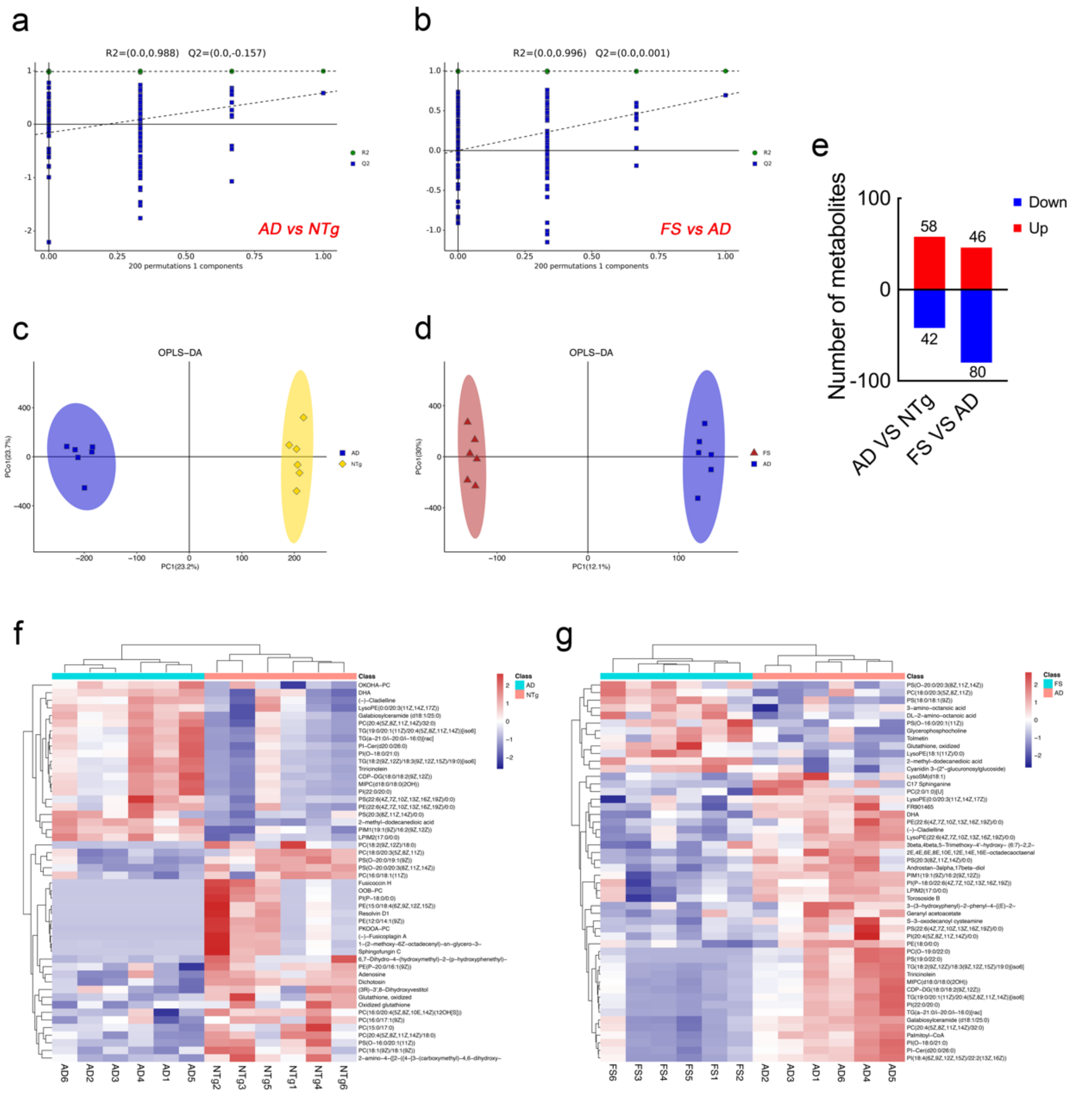

Figure S2 **a**, **b** Response permutation testing of OPLS-DA models (**a** AD vs. NTg, **b** FS vs. AD). **c**, **d** Score plots accurately distinguished NTg, AD and FS by OPLS-DA analysis (**c** AD vs. NTg, **d** FS vs. AD). **e** Number of upregulated and downregulated metabolites in the two paired comparisons of AD vs. NTg and FS vs. AD. **f**, **g** Clustering heatmap of the top 50 differential metabolites in the comparison groups of AD vs. NTg (**f**) and FS vs. AD (**g**).

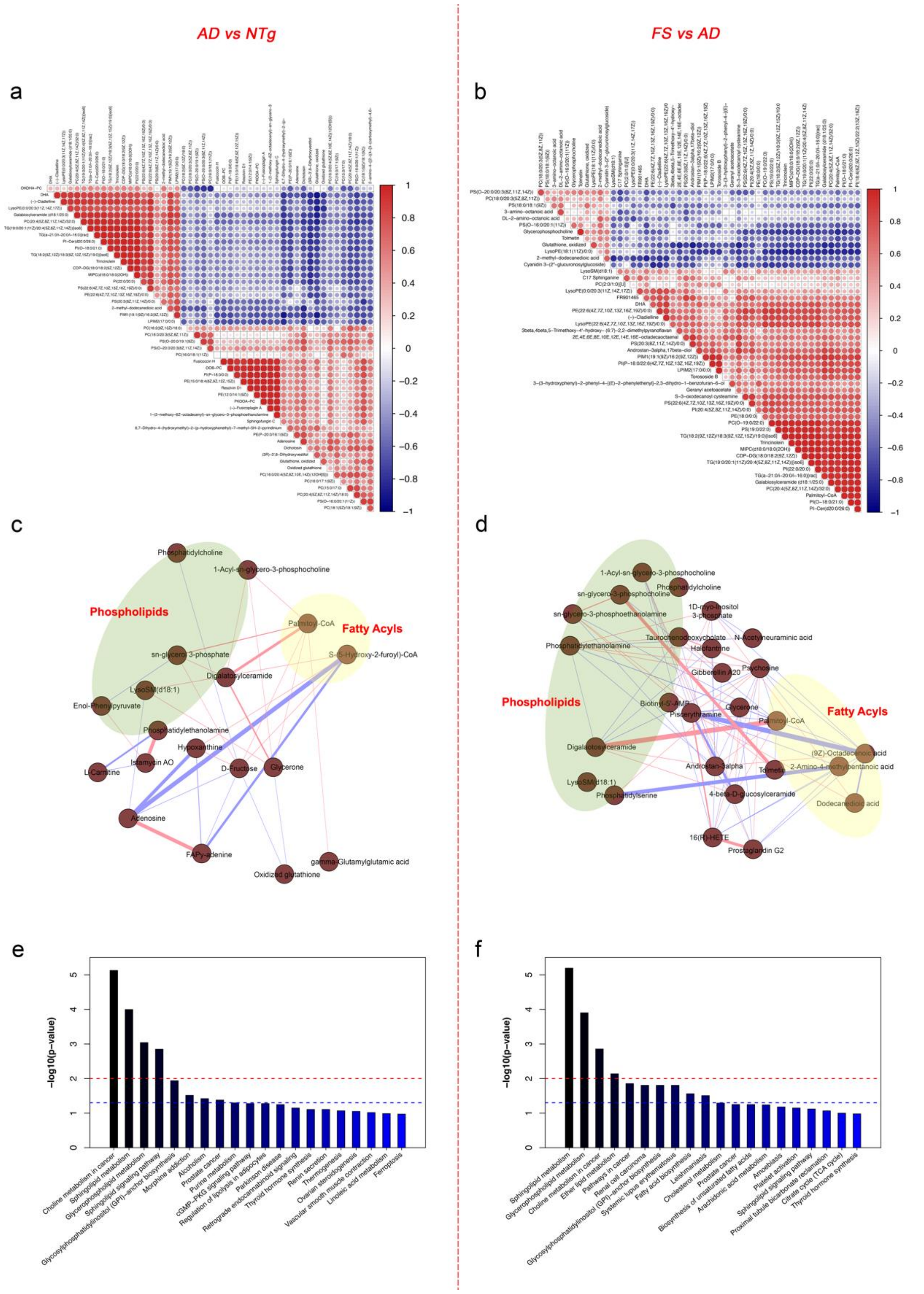

Figure S3 **a**, **b** Correlation of the top 50 differential metabolites from the Pearson correlation coefficient analysis of the two paired comparisons (**a** AD vs. NTg, **b** FS vs. AD). **c**, **d** The functional correlation network of differential metabolites in the KEGG database (**c** AD vs. NTg,

d FS vs. AD). |r|>0.7. **e**, **f** KEGG enrichment analysis of differential metabolites. (**e** AD vs. NTg, **f** FS vs. AD).

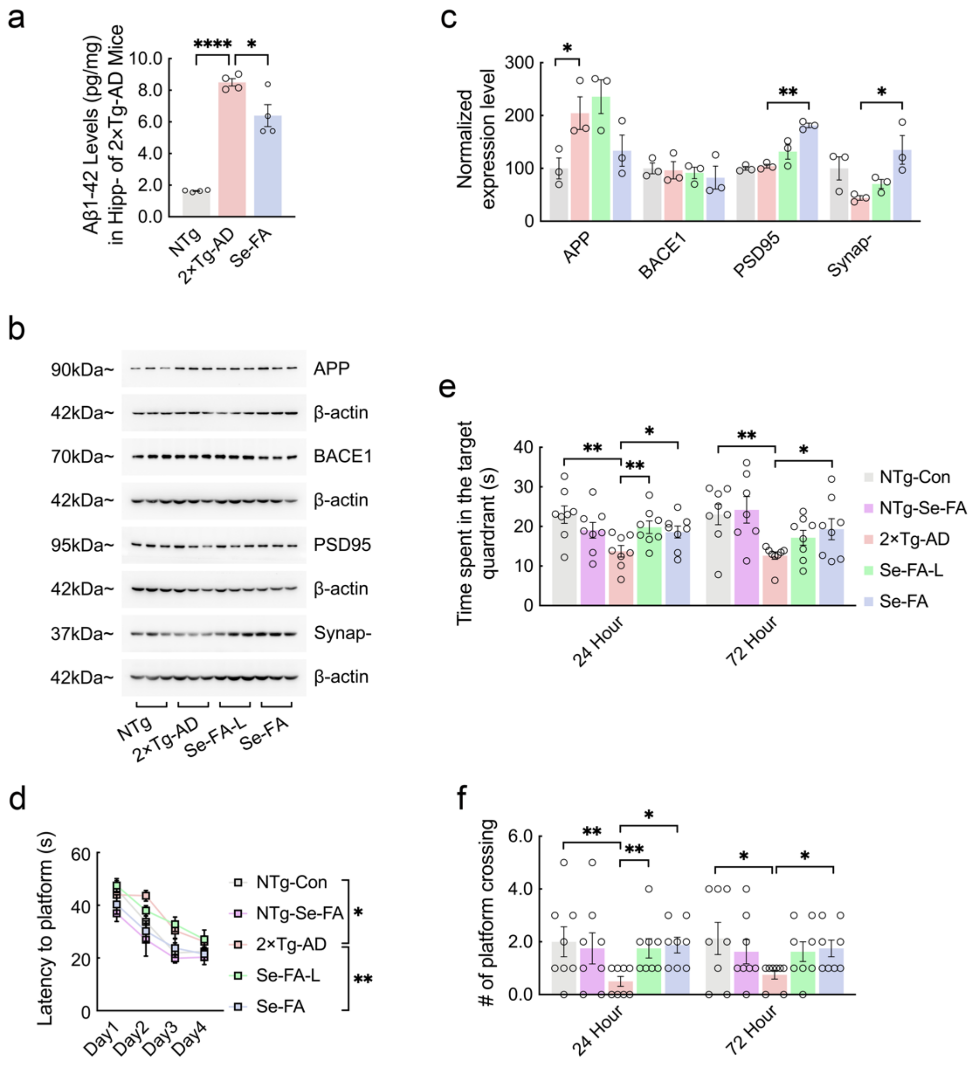

Figure S4 a Aβ1-42 levels in the hippocampus of 7-month-old 2×Tg-AD mice measured by ELISA (n=4 mice). **b** Levels of APP, BACE1, PSD95 and synaptophysin were analyzed by immunoblotting in the hippocampus of 2×Tg-AD mice. **c** Quantitation of the protein levels in **b** (n=3 mice). **d-f** Morris water maze tests were performed in 7-month-old 2×Tg-AD mice (n=8 mice, two-way ANOVA followed by Dunnett's multiple comparison test). The escape latency time to reach the hidden platform was recorded during the 4-day training period (**d**). The probe trial was performed 24 and 72 h after the last trial of a hidden platform task. The percentage of search time for each quadrant (**e**) and the number of crossings over the original platform area

were recorded (**f**). β-actin or GAPDH were used as loading controls. All data are presented as the mean ± SEM. *$p<0.05$, **$p<0.01$, ****$p<0.0001$ as determined by one-way ANOVA followed by Dunnett's multiple comparison test.
